# An Adenoviral Vector as a Versatile Tool for Delivery and Expression of miRNAs

**DOI:** 10.3390/v14091952

**Published:** 2022-09-02

**Authors:** Jonas Scholz, Patrick Philipp Weil, Daniel Pembaur, Georgia Koukou, Malik Aydin, Dorota Hauert, Jan Postberg, Florian Kreppel, Claudia Hagedorn

**Affiliations:** 1 Chair for Biochemistry and Molecular Medicine, Center for Biomedical Education and Research, School of Life Sciences (ZBAF), Faculty of Health, Witten/Herdecke University, 58453 Witten, Germany; 2 Centre for Biomedical Education & Research (ZBAF), Clinical Molecular Genetics and Epigenetics, Faculty of Health, Witten/Herdecke University, Alfred-Herrhausen-Str. 50, 58448 Witten, Germany; 3 Laboratory of Experimental Pediatric Pneumology and Allergology, Center for Biomedical Education and Research, School of Life Sciences (ZBAF), Faculty of Health, Witten/Herdecke University, 58448 Witten, Germany

**Keywords:** miRNA, delivery, adenoviral vectors, expression platform

## Abstract

Only two decades after discovering miRNAs, our understanding of the functional effects of deregulated miRNAs in the development of diseases, particularly cancer, has been rapidly evolving. These observations and functional studies provide the basis for developing miRNA-based diagnostic markers or new therapeutic strategies. Adenoviral (Ad) vectors belong to the most frequently used vector types in gene therapy and are suitable for strong short-term transgene expression in a variety of cells. Here, we report the set-up and functionality of an Ad-based miRNA vector platform that can be employed to deliver and express a high level of miRNAs efficiently. This vector platform allows fast and efficient vector production to high titers and the expression of pri-miRNA precursors under the control of a polymerase II promoter. In contrast to non-viral miRNA delivery systems, this Ad-based miRNA vector platform allows accurate dosing of the delivered miRNAs. Using a two-vector model, we showed that Ad-driven miRNA expression was sufficient in down-regulating the expression of an overexpressed and highly stable protein. Additional data corroborated the downregulation of multiple endogenous target RNAs using the system presented here. Additionally, we report some unanticipated synergistic effects on the transduction efficiencies in vitro when cells were consecutively transduced with two different Ad-vectors. This effect might be taken into consideration for protocols using two or more different Ad vectors simultaneously.

## 1. Introduction

Micro RNAs (miRNAs) are a class of endogenous small RNAs (~22 bp). Targeting the 3′-UTR of an mRNA, these small RNA molecules can function as post-transcriptional regulators of gene expression [1,2,3] but have also been shown to be involved in transcriptional gene silencing in yeast [4,5], *Arabidopsis* [6,7], nematodes [8,9], *Drosophila* [10], and mammalian cells [11,12]. The first miRNA was discovered in 1993 in the nematode *Caenorhabditis elegans.* Downregulation of the heterochronic gene lin-14 was found to be dependent on the transcription of a second gene (lin-4) that itself was not translated [1,2]. Genes encoding miRNAs are transcribed by RNA polymerase II. Consequently, primary miRNAs (pri-miRNA) exhibit 5′-7-methylguanosine caps and 3′ poly(A) tails. A multiprotein complex containing an RNAse III enzyme, drosha, cleaves the pri-miRNA at the base of the characteristic hairpin structure. This results in a two nucleotide 3′-overhang [13,14]. The pre-miRNA is subsequently exported to the cytoplasm, where it is further processed to 18–23 bp long duplexes by the RNAse III endonuclease Dicer [15,16]. This processing step includes the removal of the terminal loop, resulting in a mature miRNA duplex [17]. Finally, the two miRNA strands are separated. The guiding strand associates with a member of the argonaute (Ago) family of proteins, forming an RNA-induced silencing complex (RISC) [18,19,20]. The human genome encodes four different Argonaut proteins (Ago 1–4) capable of loading miRNAs. Of those, Ago2 and, recently, Ago3 are described to possess endonuclease activity resulting in mRNA target cleavage [21,22]. A full complementary miRNA:mRNA interaction induces Ago2 endonuclease activity leading to mRNA cleavage [20,23]. However, interaction with perfectly complementary target sites destabilises the miRNA:RISC complex, promoting miRNA degradation [24]. Most endogenous miRNA:mRNA interactions are not entirely complementary, thus preventing the endonuclease activity of Ago2. Consequently, Ago2 mediates downstream effects similar to those induced by the non-endonucleolytic Ago family members, including the repression of translation or poly(A)-deadenylation and decapping of the target [25,26].

However, the mechanisms of miRNAs in regulating gene expression are far more complex. Besides the canonical mode of action, miRNAs have been shown to interact with other parts of a target mRNA, such as the coding sequence and 5′ UTR, or even with the gene promoter [27,28]. Additionally, a single miRNA might impact different pathways and biological processes to regulate one target gene precisely and in a finely nuanced way. Most miRNAs are therefore expressed in a temporal- and tissue-specific manner [29,30]. Recent studies reported that a large number of miRNAs derived from different tissues do exist in various body fluids, such as blood [31], urine [32], or saliva [33,34]. Due to their role in the regulation of numerous physiological processes, miRNAs have been found to be deregulated in various diseases, such as cardiovascular diseases [35,36], neurodegenerative diseases [37,38,39,40,41], autoimmune diseases [42,43,44,45], and cancer [46,47,48,49,50,51,52]. Given these observations, our understanding of miRNAs as disease markers is rapidly evolving. Understanding the functional effects of deregulated miRNAs on a cellular or systemic level may provide a basis for developing new therapeutic strategies. For example, for the treatment of hepatitis C virus infections, the miRNA-based drug Miravisen is currently in phase-II clinical trials [53]. With Patisiran, the first small-interfering RNA was granted by the FDA for the treatment of hereditary transthyretin-mediated (hATTR) amyloidosis [54,55], and also in the light of the current SARS-CoV-2 pandemic, small RNAs are extensively tested for their therapeutic potential [56]. However, highly abundant ribonucleases and sequestration by the reticuloendothelial system hinder the delivery of naked RNA molecules. The combined use of chemical modifications and lipid-based nanocarriers may increase RNA stability, including for in vivo delivery (reviewed in [57]), but are not applicable for the targeted delivery of miRNAs to specific tissues or organs other than the liver. Thus, viral delivery systems, such as adenoviral (Ad) vectors, represent an attractive alternative.

Here, we describe the set-up and functionality of an easily accessible Ad vector-based miRNA expression system. This system combines the ease and the efficiency of viral vector-mediated transduction with strong polymerase II promoter-driven miRNA expression. Importantly, and in sharp contrast to non-viral systems, it enables precise dosing and is, thus, well suited for a wide variety of in vitro research applications. Finally, we present a series of unanticipated effects that were observed after consecutive in vitro transduction of cells with two different Ad vectors. These effects might, in general, impact the experimental design of assays relying on Ad vector-based transduction.

## 2. Materials and Methods

### 2.1. Cell Lines and Cell Culture

A549 (ATCC^®^ CCL-185™) and HEK293 (ATCC^®^ CRL-1573™) cells were cultured in MEM supplemented with 10% FCS and penicillin (100 U/mL)/ streptomycin (100 µg/mL). Cells were passaged twice per week using 0.05% trypsin/ 0.02% EDTA. All cell culture products were purchased from PAN, Hamburg, Germany.

### 2.2. Vector Construction

All vectors used in this study were first-generation vectors (*E1* deleted) of human adenovirus type 5 (AY339865; bp 1-440; bp 3522-35934). Gene expression cassettes were introduced using pRed/ET homologous recombination (Gene Bridges, Heidelberg, Germany) as described below. First, a miRNA expression cassette based on pcDNA^TM^6.2-GW/EmGFP-miRNA-neg control plasmid (Invitrogen, Darmstadt, Germany) was introduced into the E1-deleted region of Ad5, resulting in pAd-miRNAscr. This cassette allows the constitutive expression of miRNAs and a co-cistronic reporter gene (EmGFP) under the control of CMV promoter. Vector pAd-miRNAscr encodes for a nonsense miRNA (miRscr: 5′-AAAUGUACUGCGCGUGGAGAC-3′) and served as negative control in all experiments. Next, the nonsense miRNA sequence was replaced with a counter-selection cassette (rpsl-neo; Gene Bridges, Heidelberg, Germany), resulting in pAdΔE1-miRNA-rpslneo. This construct allows for the subsequent replacement with any miRNA sequence of interest. For a proof-of-concept experiment, two miRNAs targeting the red fluorescent protein tdTomato were cloned into pAdΔE1-miRNA-rpslneo, resulting in pAd-Tom1 (miRTom1: 5′-UACUGUUCCACGAUGGUGUAG-3′, htttps://rnaidesigner.thermofisher.com Access date: 2 August 2022) and pAd-Tom2 (miRTom2: 5′-UUGGUGUCCACGUAGUAGUAG-3′, [58]). Ad-EGFP harbours a CMV-promoter-driven EGFP expression cassette at (AY339865, bp 441-3534). Based on Ad-EGFP, Ad-EGFP∆CAR∆FX and Ad-EGFP/MPM2K were constructed as follows: two point mutations were introduced in the fiber knob domain (AAQ19310.1, Y477A) [59] and hexon HVR7 (AAQ19298.1, T425A) [60] to ablate binding to the Coxsackievirus and adenovirus receptor (CAR) and blood coagulation factor X (FX), resulting in Ad-EGFP∆CAR∆FX. Two cysteines were introduced into the hexon HVR1 (AAQ19298.1, D151C) and HVR5 (AAQ19298.1, A273C) of Ad-EGFP to insert a chemical reactivity group for position-specific PEGylation. In the Ad-empty vector, the E1-gene region (AY339865, bp 441-3521) was deleted, and a PacI-restriction site was inserted. This vector does not contain a heterologous expression cassette. All constructs were sequenced and analysed by restriction digestion to ascertain sequence integrity. All constructs used in this study are freely available upon request.

### 2.3. Adenovirus Vector Purification

Adenoviral vectors were transfected, amplified and purified as described elsewhere [61]. Briefly, HEK293 cells, transcomplementing *E1* [62], were transfected with linearised vector DNA using polyethyleneimine (PEI) and harvested when a cytopathic effect became visible. Subsequently, vectors were amplified by sequential re-infections and purified using CsCl-gradient centrifugation. Two consecutive, discontinuous CsCl gradients were performed, followed by a desalting procedure using PD10 columns (GE Healthcare, Solingen, Germany) [61]. For cysteine-carrying vectors, lysis buffers containing 0.1 mM TCEP (Invitrogen, Darmstadt, Germany) as a reducing reagent to prevent the aggregation of particles due to the oxidation of cysteines were used [63]. Vectors were lysed with 0.5% sodium dodecyl sulfate and incubated for 10 min at 65 °C to determine physical titers by optical density at 260 nm [64].

### 2.4. PEGylation of Vector Capsids

After the first CsCl gradient, maleimide-activated bifunctional linear polyethylenglycol (PEG, 2 kDa) (MPM2K; IRIS Biotech, Marktredwitz, Germany) was used to PEGylate vector particles. Immediately before mixing with vectors, PEG moieties were dissolved as a 10% (*w*/*v*) solution in Ad-buffer (50 mM HEPES, 150 mM NaCl, pH 7.2). Vectors were incubated with PEG solution (final concentration 1.67%; about 2000-fold PEG excess over cysteines), gently rotating for 1 h at room temperature. Subsequently, Ad-EGFP/MPM2K vectors were purified by a second discontinuous CsCl gradient centrifugation followed by PD10 column desalting [65] to remove uncoupled PEG moieties.

### 2.5. Polymerase Chain Reaction (PCR)

All DNA fragments used for homologous recombination were amplified using Q5 High-Fidelity polymerase (New England Biolabs, Frankfurt, Germany) and primers sharing a 50 bp homology to the site of insertion (Table S1). Each reaction contained 0.2 mM dNTPs (Thermo Fisher, Karlsruhe, Germany), 0.5 µM primer forward and reverse, 0.02 U/µL of Q5 High-Fidelity polymerase (New England Biolabs, Frankfurt, Germany), and 1–5 ng of template DNA. PCR cycles were performed as follows: 1 cycle: 30 s 98 °C (denaturation); 26 cycles: 10 s 98 °C (denaturation)—30 s X °C (annealing, see Table S1)—3 min 72 °C (elongation); 1 cycle: 10 min 72 °C (elongation). Replacement of rpsl-neo counter-selection cassettes with a miRNA sequence of choice was ascertained by colony PCR. Clones were pre-cultured for 3 h at 37 °C in 100 µL LB medium (Invitrogen, Darmstadt, Germany) supplemented with respective selection markers. Subsequently, 3 µL of bacteria suspension was mixed with 0.2 µM primer forward and reverse (5′-GGATCACTCTCGGCATGGAC-3′ and 5′-ATTGCCGTCATAGCGCGGGT-3′), 0.2 mM dNTPs and 0.025 U/µL polymerase. PCR cycles were performed as follows: 1 cycle: 2 min 98 °C (denaturation); 33 cycles: 30 s 98 °C (denaturation)—45 s 58 °C (annealing)—2 min 72 °C (elongation); 1 cycle: 10 min 72 °C (elongation).

### 2.6. Transduction Assays

Transduction assays were performed using A549 cells. To analyse the effect of miRNA expression on its target on protein level, 1 × 10^5^ cells/well were seeded in 24-well plates and cultivated overnight. The following day, cells were transduced in triplicates with miRNA-expressing vectors Ad-miRscr, Ad-miRTom1, or Ad-miRTom2 with a multiplicity of infection (MOI) of 2000 and incubated for 12 h at 37 °C, 5% CO_2_. Subsequently, cells were transduced with Ad-tdTomato (MOI 500, expressing the target gene) and incubated at 37 °C, 5% CO_2_. Cells were then washed three times with phosphate-buffered saline (PBS) and analysed 24, 48, 72, and 96 h post-transduction (h.p.t.) for fluorescent gene expression using flow cytometry (CytoFlex, Beckman Coulter, Munich, Germany). A 488 nm laser was used for excitation, EGFP expression was analysed in an FITC channel (525/40 nm), and tdTomato expression was analysed in a PE-channel (585/42 nm).

5 × 10^5^ cells/well were seeded in 6-well plates and cultivated overnight to monitor Ad-driven miRNA expression over time. On the next day, cells were transduced in triplicates with miRNA-expressing vectors Ad-miRscr, Ad-miRTom1, and Ad-miRTom2 (MOI 500). Total RNA was extracted 24, 48, 72, and 96 h.p.t. as described below. To analyse target gene mRNA level, 1 × 10^6^ cells/well were seeded in 6-well plates and cultivated overnight. The following day, cells were transduced with an overall MOI of 2500, containing different amounts of Ad-miRTom1 (MOI 10, 100, 1000, 2500) complemented with a non-expressing vector (Ad-empty). Six hours after first transduction, cells were transduced with Ad-tdTomato at MOI 100, incubated for 18 h, and total RNA was extracted as described below.

To quantify Ad-genomes in the cells after transduction, 3 × 10^5^ cells/well were seeded in 12-well plates and cultivated overnight. The next day, cells were transduced with a non-expressing vector (Ad-empty) at different MOIs (MOI 10, 100, 1000, 2500) and incubated for six hours. Subsequently, cells were transduced with EGFP-expressing vectors Ad-EGFP, Ad-EGFP∆CAR∆FX, and Ad-EGFP/MPM2K (MOI 100) and incubated for 18 h. Total genomic DNA was extracted as described below and used as a template in qPCR analyses.

### 2.7. Isolation of Total RNA and cDNA Synthesis

Cells grown in 6-well plates were harvested in 1 mL Trizol (Invitrogen, Darmstadt, Germany) per well and stored at −80 °C or directly processed according to the manufacturer’s instruction. Prior to cDNA synthesis, 10 µg of extracted total RNA was treated with 2.5 U DNAse (35 min, 37 °C) in a total volume of 50 µL. Subsequently, 3.75 µL DNAse-treated RNA was subjected to polyadenylation and reverse transcription using a Mir-X miRNA First-Strand Synthesis Kit (Takara Bio, Göteborg, Sweden). The reaction was performed in a thermo cycler for 60 min at 37 °C in a total volume of 10 µL. Newly synthesised cDNA was diluted with 90 µL ultra-pure DNase/RNase-free water and either stored at −80 °C or subsequently used as a template in quantitative PCR analyses.

### 2.8. Isolation of Total Genomic DNA

Cells were detached with trypsin and collected by centrifugation at 300 × *g* for 10 min. The pellet was resuspended in 200 µL PBS and mixed with 200 µL lysis buffer (10 mM Tris, 10 mM EDTA, 0.5% SDS). Proteinase digestion was performed overnight at 50 °C in the presence of 0.9% SDS and 400 µg proteinase K. The next day, samples were treated with 200 µg RNAse A (30 min, 37 °C). Subsequently, DNA was isolated using phenol-chloroform extraction followed by ethanol precipitation. DNA concentration was estimated by measuring the absorbance at 260 nm, and concentrations were adjusted to 5 ng/µL prior to subjecting to qPCR analyses.

### 2.9. Real-Time Quantitative PCR Analysis

Expression levels of transduced miRNAs and targeted mRNA were evaluated in quantitative PCR analyses. For quantification of the miRNA level, 2 µL of cDNA were mixed with 10 µL TB Green Advantage Premix (Takara Bio, Göteborg, Sweden), 0.2 µM primer forward and reverse (EGFP: forward 5′-CGACCACTACCAGCAGAACA-3′, reverse 5′-GAACTCCAGCAGGACCATGT-3′; U6 snRNA (Takara Bio, Göteborg, Sweden), miRscr: forward 5′-AAATGTACTGCGCGTGGAGAC-3′; miRTom1: forward 5′-TACTGTTCCACGATGGTGTAG-3′; miRTom2: forward 5′- TTGGTGTCCACGTAGTAGTAG-3′; mRQ 3′ Primer (Takara Bio, Göteborg, Sweden) served as a universal reverse primer) in a final volume of 20 µL. PCR cycles were performed as follows: 1 cycle: 1 min 95 °C (denaturation); 45 cycles: 10 s 95 °C (denaturation)—25 s 60 °C (annealing/elongation). 

Target mRNA was quantified by mixing 2 µL cDNA with 10 µL TB Green Advantage Premix (Takara Bio, Göteborg, Sweden), 0.2 µM primer forward and reverse (tdTomato: forward 5′-ACCTGTTCCTGGGGCATG-3′; reverse 5′-TGATGACGGCCATGTTGTTG-3′) in a final volume of 20 µL. PCR cycles were performed as follows: 1 cycle: 10 min 95 °C (denaturation); 45 cycles: 15 s 95 °C (denaturation)—30 s 60 °C (annealing)—30 s 72 °C (elongation). Relative RNA expression levels were calculated using the ΔΔCt method. All samples were normalised to U6 snRNA and subsequently compared with cells either not treated with Ad-miRNA (“w/o”; tdTomato mRNA level) or the lowest MOI used for transduction (“MOI 10”, miRNA expression level).

To quantify Ad genomes in transduced cells, 20 ng of total genomic DNA was mixed with 10 µL TB Green Advantage Premix (Takara Bio, Göteborg, Sweden), 0.2 µM primer forward and reverse (CMV: forward 5′-TACATCAATGGGCGTGGATA-3′, reverse 5′-GGCGGAGTTGTTACGACATT-3′; plasminogen activator tissue type (PLAT): forward 5′-AGGGCTGGAGAGAAAACCTC-3′, reverse 5′-TTCCTTCACTGGCTCAGCTT-3′) in a final volume of 20 µL. PCR cycles were performed as follows: 1 cycle: 10 min 95 °C (denaturation); 40 cycles: 30 s 95 °C (denaturation)—45 s 60 °C (annealing/ elongation). The number of Ad genomes per ng DNA was calculated using the ΔΔCt method. All samples were normalised to PLAT and subsequently compared to samples that were transduced in the absence of Ad-empty. 

### 2.10. Statistical Analysis

Experiments were carried out in independent replicates with a minimum of *n* = 3. Statistical analyses were performed with RStudio. First, data were tested for normality using the Shapiro Wilk test. Testing for homogeneity of variances was performed using Levene’s Test. When data met the criterium of variance homogeneity, means were tested for significant differences using one-way ANOVA followed by either TukeyHSD PostHoc test or the Dunnett Test for many-to-one comparisons. In the case of non-homogeneity, ANOVA with Welch correction and the Games-Howell PostHoc test were applied.

## 3. Results

**Figure 1:**
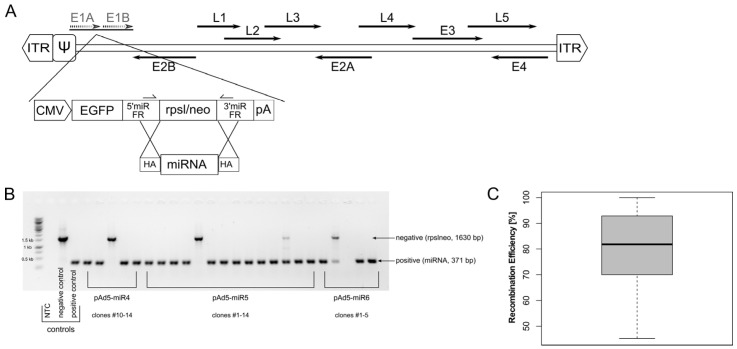
An adenoviral vector used for expression of miRNAs. (**A**) An expression cassette encoding for EGFP reporter gene, and a pre-miRNA driven by a CMV promoter was inserted in E1-deleted region of Ad5. Subsequently, miRNA sequence was replaced with a counter-selection marker (rpsl/neo) enabling fast and efficient insertion of any miRNA of choice via homologous recombination. Primer located in 5′- and 3′-miR flanking regions were used to screen for positive clones after recombination. (**B**) Primers located within the 5′- and 3′-miR flanking region were employed to screen for miRNA insertion in a diagnostic colony PCR. Constructs harbouring the rpsl/neo counter selection cassette resulted in a 1630-bp PCR fragment, whereas successful miRNA insertion resulted in a 371-bp PCR. Ad-miRNA-rpslneo and Ad-miRscr served as negative and positive control, respectively. (**C**) Recombination efficiency was determined by inserting 13 different miRNAs; number of positive clones was ascertained with PCR. On average, miRNAs could be inserted with an efficiency of 80.6 ± 16.7%. ITR, inverted terminal repeats; ψ, packaging signal; E1–E4, early genes; L1–L5, late genes; 5′-/3′- miR FR, 5′-/3′- miRNA flanking regions; HA, homology arms; NTC, no template control.

In this study, we set up an easily accessible and efficient adenoviral vector platform for miRNA expression. A bi-cistronic expression cassette encoding for a double-stranded pri-miRNA of interest and a reporter gene (EGFP) under the control of a constitutive polymerase II promoter (CMV) was inserted into the *E1*-deleted region of Ad5 (Figure 1A) [66]. The sequence encoding for nonsense miRNA (miRscr) was replaced with a counter-selection marker (rpsl/neo). This construct served as a platform for the insertion of different miRNAs. To demonstrate the straightforwardness of this cloning strategy, we have inserted 13 different miRNAs and screened for positive clones using diagnostic primers located within the 5′- and 3′-miRNA-flanking regions (5′-/3′-miR FR, Figure 1A). In contrast to screening approaches based on restriction enzymes, the PCR screening assay enabled the fast and accurate identification of correct clones (Figure 1B) and revealed a high insertion efficiency. Correct clones were obtained with an efficiency of 80.6% (±16.7%) (Figure 1C, Table 1).

**Figure 2:**
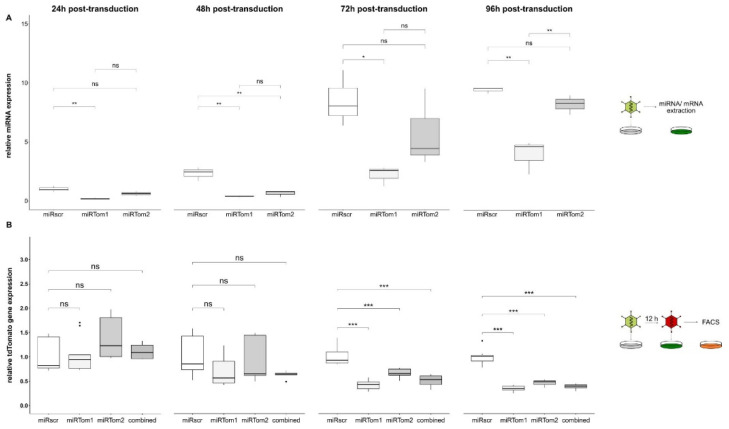
Ad-derived miRNAs were stably expressed for 96h and suppress target gene expression. (**A**) We monitored Ad-driven miRNA expression (MOI 500) over 96 h using quantitative PCR. Control miRNA miRscr showed elevated expression levels compared to miRTom1 and miRTom2 at all time points. Further, all miRNAs seemed to accumulate in the cell over time (24 h vs. 96 h: miRscr, 9,51-fold; miRTom1, 3.99-fold; miRTom2, 8.28-fold). (**B**) A549 cells were transduced with either Ad-miRTom1, Ad-miRTom2, or both vectors in combination; Ad-miRscr served as negative control (MOI 2000). Cells were then transduced with Ad-tdTomato (MOI 500) with delay of 12 h. Target gene expression (tdTomato) was normalised to miRNA-expression (EGFP), and changes in tdTomato gene expression were calculated relative to Ad-miRscr-treated cells. Target gene expression remained unaffected within the first 48 hpt (miRTom1: 0.69 ± 0.29, *p* = 0.083; miRTom2: 0.91 ± 0.42, *p* = 0.54; combined: 0.63 ± 0.07, *p* = 0.068), but significantly decreased 72 hpt with a reduction of 57.7% ± 9.31% for miRTom1 (miRTom1: 0.42 ± 0.09, *p* < 0.001; miRTom2: 0.66 ± 0.09, *p* < 0.001; combined: 0.51 ± 1.2, *p* < 0.001). This suppression became more distinct at 96 hpt. Overall, miRTom1 achieved a reduction of 65.9% ± 6.1% and thus proved to be most potent in target down-regulation (miRTom1: 0.34 ± 0.06, *p* < 0.001; miRTom2: 0.46 ± 0.06, *p* < 0.001; combined: 0.38 ± 0.06, *p* < 0.001). Significance values were calculated with Dunnett’s test. MFI, mean fluorescence intensity; hpt, hours post-transduction; ns, *p* > 0.05; *, *p* < 0.05; **, *p* < 0.01; ***, *p* < 0.001.

To prove the vector platform’s applicability, we established a two-vector system for miRNA-targeting validation. Two miRNAs targeting the red fluorescent protein tdTomato were tested, and a nonsense-miRNA (miRscr) served as a control. The resulting vectors Ad-miRTom1, Ad-miRTom2, and Ad-miRscr were used to determine the miRNA-expression levels exemplarily, both in a dose-dependent and time-dependent manner. Further, their potency of target gene downregulation was analysed. The expression of target mRNA and all three miRNAs (miRscr, miRTom1, and miRTom2) were analysed 24, 48, 72, and 96 h post-transduction (hpt). MicroRNA-expression levels were normalised to miRscr levels at 24 hpt. Surprisingly, distinct differences in miRNA expression were noticed. Overall, nonsense miRNA miRscr showed elevated expression levels compared to miRTom1 and miRTom2 at all time points (Figure 2A), while no significantly different expression levels were observed for miRTom1 and miRTom2 at 24, 48, and 72 hpt. However, all miRNAs seemed to accumulate in the cell over time, with a 9.5 ± 0.26-fold increase in miRNA abundance for miRscr and a 13.6 ± 1.36-fold increase in miRNA abundance for miRTom2 at 96 hpt. Although miRTom1 showed comparable low expression levels, an increased accumulation compared to miRscr and miRTom2 (96 hpt: 21.3 ± 7.73-fold increase over 24 hpt) was detected. In contrast, only minimal oscillation of EGFP mRNA-level between the different miRNA-expressing vectors and time points was detected (Figure S1).

However, it was questionable whether one copy of miRNA sequence per vector genome was sufficient for efficient miRNA expression and targeting. No significant effect of miRNA expression on target tdTomato was detected at 24 and 48 hpt. In contrast, 72 hpt tdTomato expression was significantly reduced in cells treated with either Ad-miRTom1 (reduction by 57.7% ± 9.31%), Ad-miRTom2 (reduction by 34.02% ± 9.16%), or both vectors combined (reduction by 48.8% ± 12.4%). This effect became more distinct at 96 hpt. Overall, Ad-miRTom1 reduced tdTomato expression by 65.9% ± 6.1% and thus proved to be the most potent in target down-regulation (Figure 2B). To confirm the potency of the system in downregulating endogenous targets, a cattle-specific miRNA with possible targets within the human genome was delivered to human fetal intestinal epithelial cells (HIEC-6). Subsequent transcriptome analyses revealed an overall downregulation of many of the 519 predicted target transcripts (0.9587-fold; *p* = 0.0032). Six target mRNAs were significantly downregulated with a fold-change of less than 0.5 [67] (Figure S2). These data also indicate the suitability of the Ad system for cell-endogenous targets.

**Figure 3:**
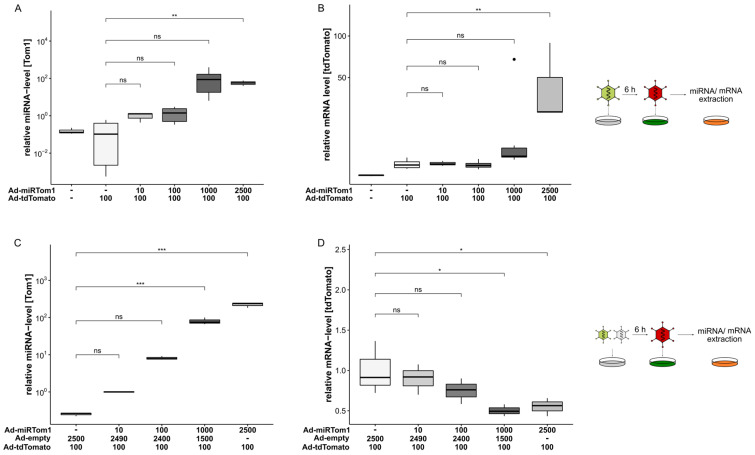
Dose-dependent expression of miRNA and targeting. A549 cells were transduced with Ad-tdTomato (MOI 100) and escalating MOIs of Ad-miRTom1. Expression levels of miRTom1 and target RNA were analysed in quantitative PCR. (**A**,**C**) Expression of CMV-driven miRNA (miRTom1) was scalable and correlated with physical adenoviral vector particles used for transduction (C: MOI 100: 9.7-fold expression over MOI 10; MOI 1000: 72.4-fold expression over MOI 10; MOI 2500: 220.2-fold expression over MOI 10; r^2^ = 0.98). (**B**) Escalating doses of Ad-mirTom1 resulted in increased mRNA levels for tdTomato (44.5 ± 39.9, *p* = 0.006). (**D**) Complementation of applied physical Ad-miRTom1 vector particles with Ad-empty to an MOI 2500 resulted in dose-dependent downregulation (up to 50% reduction, *p* = 0.02) of target mRNA. Significance values were calculated with Dunnett’s test. MOIs (multiplicity of infection) were applied as indicated. ns, *p* > 0.05; *, *p* < 0.05; **, *p* < 0.01; ***, *p* < 0.001.

For further characterisation of this two-vector system and to demonstrate its scalability, miRNA expression and target mRNA suppression were analysed in a dose-dependent manner. Although it had a comparable low expression level (Figure 2A), miRTom1 proved to most efficiently suppress tdTomato expression at the protein level (Figure 2B) and was thus employed for further characterisation. Since miRTom1 exhibited a full complementary miRTom1:tdTomato interaction, it is conceivable that upon binding to its target, Ago2 endonuclease activity was induced, resulting in tdTomato mRNA cleavage. Therefore, the A549 cells were transduced with different MOIs of Ad-miRTom1, followed by transduction with Ad-tdTomato. Expectedly, the expression of miRTom1 was scalable and correlated with the applied particle titer (Figure 3A). However, the mRNA level of tdTomato surprisingly increased with escalating doses of Ad-miRTom1. A 44.5 ± 39.9-fold (*p* = 0.006) increase in tdTomato mRNA level was observed when the cells were pre-treated with Ad-miRTom1 (MOI 2500, Figure 3B). To analyse the potential mechanism underlying this increase, we transduced the cells with an overall MOI of 2500, containing different amounts of Ad-miRTom1 complemented with a non-expressing vector (Ad-empty), followed by delayed transduction with Ad-tdTomato. Again, the miRTom1 expression levels were scalable and correlated with the applied particle titer (r^2^ = 0.98, Figure 3C). Furthermore, the mRNA levels decreased dose-dependently, resulting in a reduction in tdTomato mRNA by 50% in the cells treated with Ad-miRTom1 (0.5 ± 0.07, MOI 1000) compared to the untreated cells (1.0 ± 0.33; Figure 3D). These data demonstrate that the Ad-derived miRNA expression levels were sufficient for target suppression involving miRNA-directed mRNA cleavage. Moreover, these data suggest synergistic effects on Ad vector transduction in multiple vector approaches.

**Figure 4:**
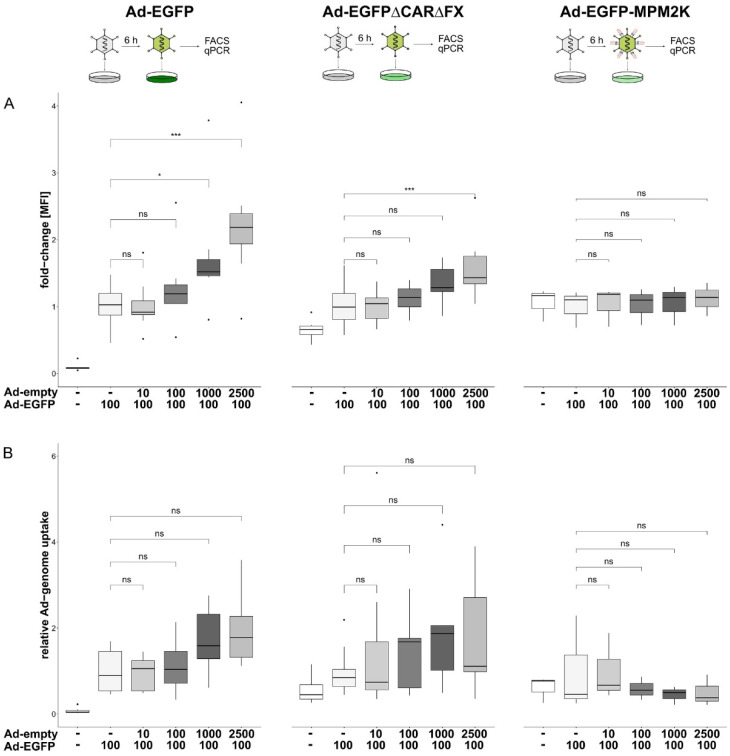
Effects of Ad-vector pre-treatment on vector particle uptake and cellular trafficking. Cells were transduced (pre-treated) with escalating doses of Ad-empty followed by transduction with EGFP-expressing vectors harbouring different capsid-modifications (left panel, wt capsid; center, ablated binding of CAR and FX; right panel, irreversible coupling to bi-functional PEG MPM2K). (**A**) 24 hpt transgene expression was analysed in flow cytometry. Increasing doses of Ad-empty resulted in increased transgene expression of Ad-EGFP (2.22 ± 0.91-fold, *p* < 0.001) and Ad-EGFP∆CAR∆FX (1.56 ± 0.48-fold, *p* < 0.001). However, transgene expression was not detected for Ad-EGFP-MPM2K (1.11 ± 0.24-fold, *p* = 0.634). (**B**) Genomic DNA at 24 hpt was extracted and EGFP-containing vector genomes were quantified. Again, escalating doses of Ad-empty correlated with an increased number of EGFP-containing vector genomes per cell for Ad-EGFP (1.99 ± 0.92-fold, *p* = 0.056) and Ad-EGFP∆CAR∆FX (2.24 ± 1.95-fold, *p* = 0.083), although this was not statistically significant. However, genome copies of Ad-EGFP-MPM2K could not be detected (0.5 ± 0.36-fold, *p* = 0.98). Significance values were calculated with Dunnett’s test. MOIs (multiplicity of infection) were applied as indicated. MFI, mean fluorescence intensity; ns, *p* > 0.05; *, *p* < 0.05; ***, *p* < 0.001.

To determine whether the initially observed increase in target mRNA resulted from either a dose-dependent increase in particle uptake or alterations in intracellular trafficking, cells were transduced with different MOIs of a non-expressing vector (Ad-empty) followed by transduction with EGFP-expressing vectors harbouring different capsid modifications. The genome copies per cell and transgene expression were determined by qPCR and flow cytometry to distinguish between the vector uptake into the cell and transport to the nucleus. Pre-treatment of the cells with an increasing number of Ad-empty particles resulted in an increased expression of the transgene EGFP that was delivered with a wildtype (wt) capsid Ad vector (Ad-EGFP; 2.2-fold ± 0.9, *p* < 0.001; Figure 4A, left panel). A similar effect was observed for an EGFP-expressing vector ablated for binding to its natural Coxsackievirus and adenovirus receptor (CAR) and factor X (FX). Two point mutations located in the fiber knob (Y477A, ∆CAR) and hexon (T425A, ∆FX) hinder vector entry via CAR and heparan-sulfate-proteoglycans (HSPGs), respectively [68,69,70]. As such, compared to the wt-capsid Ad-EGFP, the overall transduction efficiency of Ad-EGFP∆CAR∆FX was decreased (Figure S3). However, comparing the transduction efficiencies of Ad-EGFP∆CAR∆FX concerning escalating Ad-empty doses revealed a similar effect as observed for Ad-EGFP. A pre-transduction with an increasing number of Ad-empty particles subsequently resulted in an increase in the transgene expression of Ad-EGFP∆CAR∆FX (1.56-fold ± 0.48, *p* < 0.001; Figure 4A, center). A shielded vector covalently coupled with a maleimide (mal)-activated bifunctional polymer served as a “trafficking control”. This geneti-chemically modified vector was coupled with mal-PEG(2K)-mal (MPM2K) via cysteines introduced in the hexon hypervariable regions 1 and 5 (HVR1/5). This shielding is irreversible and thus expected to interfere with cellular trafficking [71]. Accordingly, EGFP expression was completely abolished in cells transduced with Ad-EGFP-MPM2K, both in the absence and presence of Ad-empty (1.1-fold ± 0.24, *p =* 0.634; Figure 4A, right panel). Next, we quantified the relative amount of Ad vector genomes to analyse whether the observed increase in transgene expression resulted from an enhanced uptake of vector genomes. Here, a slight increase in vector particle uptake upon Ad-empty treatment was observed for Ad-EGFP (1.99-fold ± 0.9, *p* = 0.056; Figure 4B, left panel) and Ad-EGFP∆CAR∆FX (2.24-fold ± 1.95, *p* = 0.08; Figure 4B, center). However, these effects were not statistically significant. Surprisingly, Ad-EGFP-MPM2K vector genomes were not detectable in the transduced cells irrespective of preceding Ad-empty transduction (0.5-fold ± 0.36, *p* = 0.98; Figure 4B, right panel).

**Figure 5:**
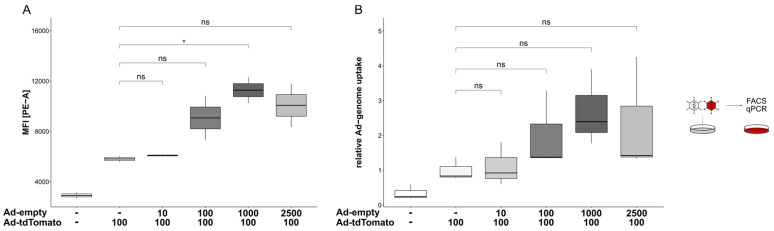
Enhancement effect of pre-treatment did not require a time-shift. Cells were transduced simultaneously with Ad-tdTomato (MOI 100) and escalating doses of Ad-empty. (**A**) Transgene expression at 24 hpt was analysed in flow cytometry. An increased expression could be detected in correlation with increased particle titers of Ad-empty (1.94 ± 0.25-fold, *p* = 0.021). (**B**) Ad-tdTomato vector genomes were quantified 24 hpt in qPCR. In a similar pattern, although not statistically significant, escalating doses of Ad-empty correlated with an elevated number of Ad-tdTomato vector genomes per cell (2.69 ± 1.1-fold, *p* = 0.09). Significance values were calculated with Dunnett’s test. MOIs (multiplicity of infection) were applied as indicated. MFI, mean fluorescence intensity; hpt, hours post-transduction; ns, *p* > 0.05; *, *p* < 0.05.

These results indicate that transduction efficiencies did not solely depend on the MOI applied. Rather, the overall quantity of the applied vector particles synergistically increased transduction efficiencies, presumably by promoting the uptake of viral particles. A similar synergistic effect was also observed when the cells were transduced simultaneously with Ad-tdTomato (MOI 100) and escalating doses of Ad-empty (MOI 1000: 1.94 ± 0.25-fold, *p* = 0.021; Figure 5A). A comparable pattern occurred when Ad-tdTomato genomes were quantified; genome copies increased with increasing doses of Ad-empty, although this was not statistically significant (2.69 ± 1.1-fold, *p* = 0.09; Figure 5B). Therefore, the observed synergistic effect on the transduction efficiencies in a multiple-vector system did not require a time offset.

## 4. Discussion

Approximately two decades after RNA interference was described in mammalian cells, three siRNA drugs have been approved, and seven others are in clinical studies [72]. Furthermore, in pre-clinical studies, small RNAs are increasingly being noticed as prognostic markers for cancers or putative therapeutic agents. The delivery of small RNAs in vitro and in vivo usually relies on lipid- or polymeric-based delivery systems, providing several advantages such as biocompatibility, high packaging capacity, and low immunogenicity [73]. However, since lipid nanoparticles used in the delivery formulations of approved siRNA drugs are homed to the liver, disease targets are usually restricted to be liver-specific. Site-specific delivery to organs other than the liver remains a significant challenge in siRNA drug development. Hence, a delivery system with the ability to efficiently target different cell and tissue types would be favorable. In this study, we constructed an adenoviral vector platform for the delivery and expression of small RNAs. Based on homologous recombination, this vector platform enabled fast and highly efficient insertion of miRNAs of interest. Thus, this non-time-consuming approach resulted in short production times and allowed miRNA insertion, vector amplification, and purification within four weeks. RNAi molecules can be delivered in different precursor formats. For cytoplasmic processing into mature silencing duplexes, the RNA molecules should be transcribed in at least a short hairpin-like structure that can be recognized by exportin 5 and Dicer (e.g., shRNA or pre-miRNA precursor). Alternatively, RNAi molecules can be transcribed as long primary miRNA (pri-miRNA) that is subsequently processed by DROSHA prior to nuclear export. While pre-miRNAs and pri-miRNAs typically contain one or more mismatches in the double-stranded stem, designed shRNAs or miRNA mimics usually have a perfect sequence complementary in the stem. Using an oncolytic adenovirus armed with a human tumor-suppressor miRNA (miR-1), Brachtlova et al. recently demonstrated that different RNAi precursor formats result in different expression levels of mature miR-1. Here, delivery in a pri-miRNA precursor format yielded, on average, more than two orders of magnitude higher levels of mature miR-1 [74]. The miRNA-expressing vector platform used in this study delivered miRNAs in a pri-miRNA-like precursor format. The RNA was transcribed with an embedded stem-loop structure containing one mismatch in the stem. Further, this pre-miRNA sequence was flanked by sequences derived from mouse miR-155 transcript [75], mimicking a pri-miRNA structure. In combination with a heterologous pol II-promoter, this ensured a high expression level of any miRNA of interest.

In a proof-of-concept study, we inserted two different miRNAs targeting the fluorescent protein tdTomato and analysed their effect on the protein expression of both protein and mRNA levels. A significant reduction in tdTomato protein was detected within 72 hpt, which was most pronounced for miRTom1. Remarkably, the combination of both miRNAs (“combined”) did not result in a more efficient down-regulation than the miRNAs alone. Fluorescently labelled Ad-particles arrive at the nuclear pore within one hpt [76,77], and gene expression is usually observed 1–2 hpt [77]. Therefore, it is reasonable to assume that the delayed effect of miRNAs on tdTomato gene expression resulted from both the high expression level of tdTomato and the prolonged half-life of the fluorescent proteins [78,79]. Further, we showed that inhibition of tdTomato protein synthesis also involved mRNA cleavage or degradation. In a dose-dependent manner, tdTomato mRNA was reduced by 50% when the cells were treated with Ad-miRTom1. Hence, miRNAs transcribed from a polymerase II-controlled co-cistronic expression cassette were appropriately processed and presumably loaded in a miRNA:RISC complex. However, the Ad-vector genome encodes for virus-associated RNAs (vaRNAs). These non-coding RNAs are transcribed by polymerase III and deregulate endogenous miRNA processing by saturating Dicer and exportin-5 [80,81]. Since vaRNAs were shown to be also transcribed from E1-depleted vectors [82], an interference with vector-encoded RNAi is conceivable. Nevertheless, detected miRNA expression and targeted downregulation up to 96 hpt, possibly due to the high CMV-driven expression level. However, vaRNAs not only interfere with miRNA processing machinery but also target cellular genes. Therefore, it should be considered that the sheer presence of Ad vectors impacts the expression of cellular genes and miRNAs. Depending on the experimental setting, these possible off-target effects should be assessed individually. Since vaRNAI is dispensable for Ad vector production [83], its deletion should be considered for future vector modifications to achieve increased downregulation efficiencies in vector-mediated RNAi [84,85].

The specificity of the interactions of miRNAs with their target mRNA is critical for miRNA function and leads to discrete downstream effects. For efficient target mRNA cleavage, a perfect miRNA:mRNA base pairing is required [86]. However, the degree of miRNA:mRNA complementarity also influences miRNA:RISC complex stability. In the absence of targets, miRNA:RISC complexes are very stable, protecting the miRNA from nuclease cleavage. Binding of miRNA:RISCs to highly complementary target RNAs leads to the formation of stable miRNA:target duplexes accompanied by rapid unloading of the RNA duplex from RISC [23,24], thus allowing the re-loading of RISC [24]. These stable miRNA:target interactions lead to efficient inhibition of translation but are not necessarily associated with target cleavage [23]. Additionally, it has recently been shown that highly expressed target RNAs lead to decreased half-lives of corresponding miRNAs as a result of increased miRNA degradation due to RISC:target interaction [24,87]. Since miRTom1 exhibits a full miRNA:target complementarity, it is very likely that miRTom1:tdTomato complexes are formed, resulting, on the one hand, in efficient downregulation of tdTomato gene expression. However, on the other hand, this high miRNA:target complementary results in unloading from RISC and consequent miRNA degradation. The detection of a reduced miRTom1 level in the Ad-miRTom1/Ad-tdTomato transduced cells, compared to control miRNA (miRscr), supports this hypothesis. 

Further, treating cells with two Ad-vectors with different MOIs surprisingly revealed a synergistic effect of Ads on the transduction efficiency. In contrast to an expected down-regulating effect, escalating doses of Ad-miRTom1 resulted in an elevated mRNA level of tdTomato. This led us to the assumption that a pre-treatment of cells with Ad-miRTom1 promoted the uptake of Ad-tdTomato in a dose-dependent manner. Accordingly, when an equal quantity of vector particles was applied, a down-regulating effect of Ad-miRTom1 on tdTomato mRNA was observed. We assumed that, in a two-vector system, pre-treatment with one Ad vector might induce either an increased particle uptake or enhanced intracellular trafficking of a second Ad vector. 

To evaluate if the observed effects were based on increased particle uptake, we used an Ad vector with two point mutations ablating both CAR-binding and FX-mediated HSPG binding (Ad-EGFPΔCARΔFX). Surprisingly, in direct comparison with a vector bearing a non-ablated wildtype capsid (AdEGFP), an up to 2-fold increase in particle uptake was observed for both vectors suggesting an increased particle uptake independent of CAR/FX-HSPG binding. Even though these effects were not statistically significant in our study, we showed that even marginal alterations in Ad particle uptake may have significant consequences on transgene expression. In the context of this strong heterologous CMV promoter, even a slight increase in vector genome copy numbers is expected to elevate transgene product levels substantially. It appears likely to us that our observations were the result of a combination of vector particle uptake and other yet unidentified effects. To further evaluate whether concomitant and consecutive transduction of multiple Ad vectors has an influence on cellular trafficking, we used a geneti-chemically modified vector (Ad-EGFP/MPM2K). An irreversible coupling of maleimide-activated bi-functional PEG (MPM2K) to HVR 1 and 5 of hexon interferes with cellular trafficking, presumably by preventing the endosomal escape [71]. Surprisingly, using this vector, we neither detected intracellular vector genomes nor transgene expression. Since samples for genome quantification were obtained 18 hpt, we assume that particles that initially entered the cell, but could not escape from the endosome, were degraded or recycled to the plasma membrane [88]. These data strongly suggest that the overall number of particles that were applied to a cell did have a synergistic effect on the transduction efficiency, rendering target cells more susceptible to Ad particle entry and thus promoting the uptake of viral particles. However, the underlying mechanism remains unclear. Although deleted for viral genes E1 and E3, residual expression of viral genes has been described for first-generation Ad (FG-Ad) vectors [89]. In this context, Martina et al. demonstrated an impact of FG-Ads on cellular gene expression, involving the up-regulation of genes associated with endocytosis and intracellular transport [90]. To resolve the mechanism underlying the observed synergistic effects in future studies, vector particle uptake could directly be monitored using fluorophore-labelled vector particles and confocal microscopy [77,91]. However, this phenomenon should be considered for multiple-vector applications in clinical protocols [92].

In summary, we developed an Ad-based vector platform for the expression and functional characterisation of miRNAs. This vector platform provided fast and efficient insertion of the desired miRNAs and allowed scalable vector production to high titers. The homologous recombination approach used for vector construction further allowed seamless deletion of the reporter gene EGFP, which may be required for future therapeutic applications. EGFP has been described as cytotoxic and immunogenic [93,94] and thus might lead to undesired side effects when delivered in therapeutic applications. Ad vectors exhibit a broad tropism and efficiently infect both dividing and non-dividing cells. Therefore, our Ad-miRNA vector platform enabled the characterisation of miRNA in vitro and in vivo without switching the delivery and expression system. Given the emerging role of RNA interference in therapeutic approaches [56,72], Ad-vectors might help to overcome the current limitations in the delivery of small RNAs. In contrast to the currently used lipid nanoparticle formulations, the capsid surface of Ad-vectors can be genetically and chemically modified. This allows for several de- and re-targeting strategies to specifically deliver small RNAs to certain organs or tissues. One of the main disadvantages limiting the widespread use of Ad vectors is the powerful stimulation of adaptive and innate immune responses. Here, geneti-chemical modifications that enable position-specific shielding of the Ad capsid might help to overcome this obstacle. These shielding strategies have shown some success and proved to protect Ad vectors from interactions with blood components and to circumvent recognition and sequestration by the immune system (reviewed in [95]). Undoubtedly, to achieve tissue-specific vector targeting and transgene expression, substantial improvements in vector design are inevitable. However, with the potential of capsid-modification strategies, in combination with tissue-specific promoters, this adenoviral miRNA-expressing vector platform might in the future be developed into a universal tool to study the functional effects of specific miRNAs in vitro and in vivo and may also be used for the delivery of therapeutic RNAs.

## Figures and Tables

**Table 1:** Efficiency of miRNA insertion. Thirteen different miRNAs were inserted in Ad-miRNA-rpslneo and the obtained clones were analysed by either digestion with restriction enzymes (miR1, miR2) or colony PCR (miR3–miR13). Positive refers to clones tested for successful miRNA insertion; negative refers to clones tested for unsuccessful miRNA insertion; other refers to ambiguous results. Of the analysed clones, at least 45.5% proved to be positive, with an average cloning efficiency of 80.6 ± 16.7%. # is the number of colonies.

	Clone #				
miRNA	Analysed	Positive	Negative	Other	Efficiency [%]
miR1	2	2	0	0	100
miR2	2	2	0	0	100
miR3	14	13	0	1	92.9
miR4	14	12	1	1	85.7
miR5	14	12	1	1	85.7
miR6	14	8	1	5	57.1
miR7	14	11	1	2	78.6
miR8	10	8	1	1	80.0
miR9	10	7	3	0	70.0
miR10	10	7	2	1	70.0
miR11	10	10	0	0	100.0
miR12	11	5	6	0	45.5
miR13	11	9	1	1	81.8

## Data Availability

Not applicable.

## Supplementary Materials

The following supporting information can be downloaded at: https://www.mdpi.com/article/10.3390/v14091952/s1, Figure S1: Stable co-cistronic expression of EGFP reporter gene over 96 hpt, Figure S2: Effects of Ad5-mediated expression of bta-miR-2404-5p, Figure S3: Direct comparison of transgene expression driven from different Ad-EGFP vectors, Table S1: Primers used for PCR amplification and homologous recombination.
