# Supplementary material for: An Adenoviral Vector as a Versatile Tool for Delivery and Expression of miRNAs
Source: Viruses. 2022 Sep 2;14(9):1952. doi: 10.3390/v14091952 (PMC9504453; doi:10.3390/v14091952)
Supplement: Supplementary file 1 [file viruses-14-01952-s001.zip › viruses-1872755-supplementary.pdf]

Supplementary Material

An adenoviral vector as a versatile tool for delivery and expression of miRNAs

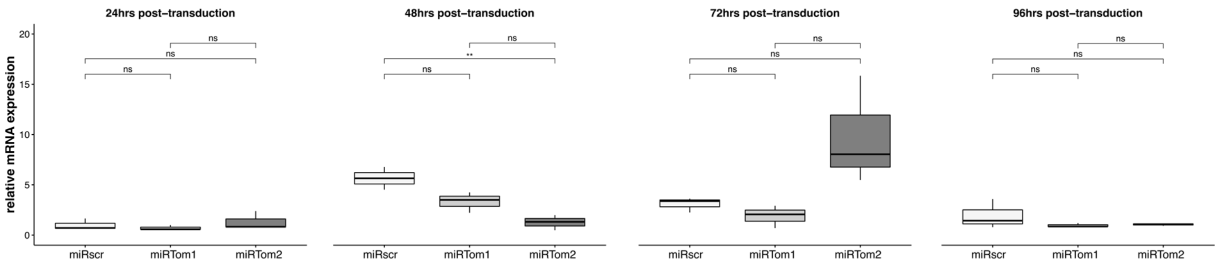

Figure S1: Stable co-cistronic expression of EGFP reporter gene over 96 hpt. We monitored Ad5dE1-driven, co-cistronic expression of EGFP over 96 h using quantitative PCR. The expression level of EGFP oscillated only slightly over time and was independent of inserted miRNA (96 hpt: miRscr 1.94-fold over 24 hpt; miRTom1 0.97-fold over 24 hpt; miRTom2 1.06-fold over 24 hpt). hpt, hours post-transduction; ns, $p > 0.05$; **, $p < 0.01$.

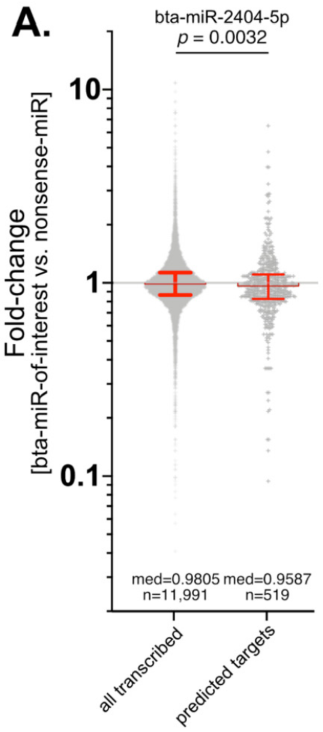

B.

>bta-miR-2404-5p
5'-UUGCACUGCAUGGUAUCUGC-3'

Gene Symbol	Gene Description	fold-change [bta-miR vs. ctrl]
ADARB2	adenosine deaminase, RNA specific B2 (inactive)	0,09415
PPARGC1A	PPARG coactivator 1 alpha	0,22056
UBE2E2	ubiquitin conjugating enzyme E2 E2	0,24608
MAB21L1	mab-21 like 1	0,44944
HEY2	hes related family bHLH transcription factor with YRPW motif 2	0,46403
DLEU7	deleted in lymphocytic leukemia 7	0,49215

Figure S2: Effects of Ad5-mediated expression of bta-miR-2404-5p. The cattle-specific miRNA bta-miR-2404-5p that does not occur in human was used to target endogenous mRNAs. Putative human targets for bta-miR-2404-5p were predicted using the 'custom prediction' option of miRDB* (http://mirdb.org/mirdb/index.html). **A.** Effects of Ad5-mediated expression of bta-miR-2404-5p were studied in HIEC-6 in comparison to HIEC-6 expressing an Ad5-mediated nonsense-miR. Relative fold-changes were compared for the whole HIEC-6 transcriptome and the lists of expressed predicted targets. Predicted targets for each bta-miR comprised of hundreds of mRNAs. Statistical power calculations were done using the nonparametric Wilcoxon–Mann–Whitney method and GraphPad Prism 8.4.3 software. **B.** Sequence of bta-miR-2404-5p. miRDB considers nucleotides 1-8 (red) for putative target interactions within the 3′-UTR of mRNAs, whereas mainly the seed region comprises of nucleotides 2-8 (underlined). Below is a list of experimentally confirmed targets exhibiting fold changes below 0.5x that were previously predicted using miRDB.

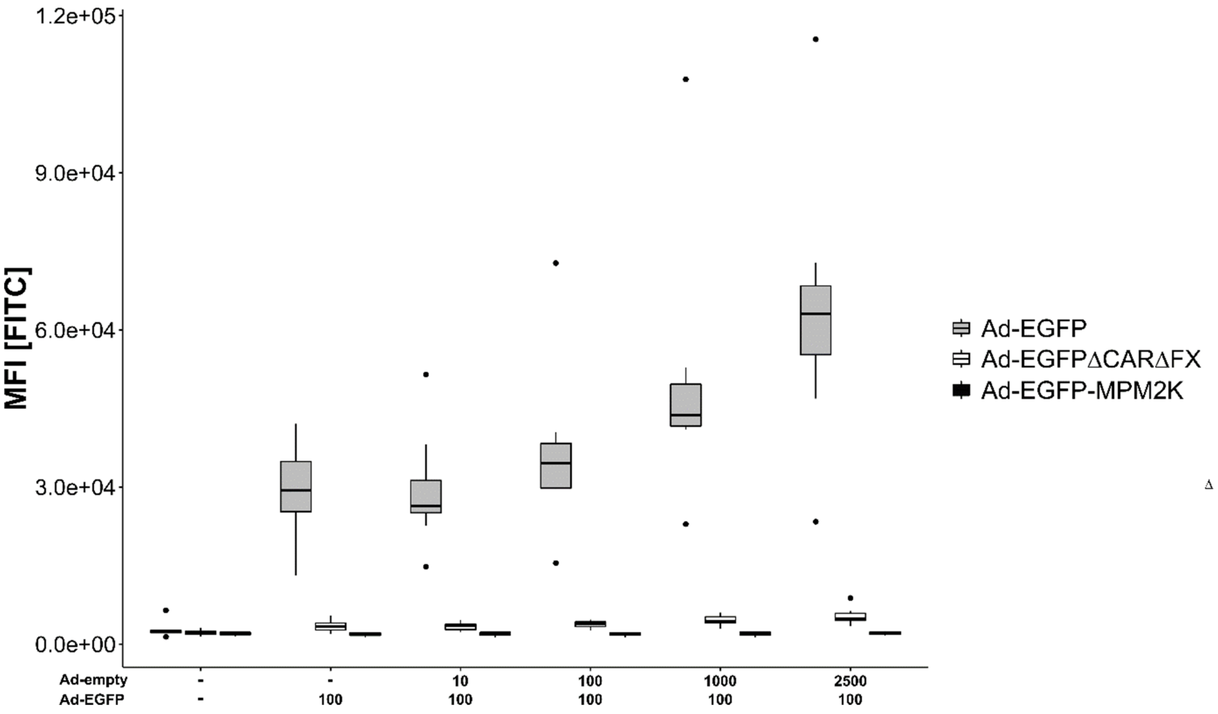

Figure S3: Direct comparison of transgene expression driven from different Ad-EGFP vectors. A549 cells were transduced with escalating doses of Ad-empty followed by transduction with an EGFP-expressing vector. Compared to Ad-EGFP, transgene expression from Ad-EGFPΔCARΔFX was significantly reduced due to its ablated binding to CAR [50]. The enhancing effect of Ad-empty co-transduction was similar for Ad-EGFP and Ad-EGFPΔCARΔFX. EGFP expression driven from Ad-EGFP/MPM2K was not detectable. MFI, mean fluorescence intensity.

Table S1 Primers used for PCR amplification and homologous recombination. Homology arms are displayed italic. Tan, annealing temperature.

target		homology arms (*italic*)	5' - 3'	Tan
miR expression cassette	forward	Ad5 bp 391-440 (AY339865)	*AGGTGTTTTTCTCAGGTGTTTTCCGCGTTCCGGGTCAAAGTTGGCGTTTT* tctgcttagggttaggcgtt	63.5 °C
miR expression cassette	reverse	Ad5 bp 3522-3571 (AY339865)	*ATAAGACCCCCACCTTATATATTCTTTCCCACCCTTAAGCCACGCCCACA* gctgccaggaaacagctatg	63.5 °C
rpslneo counter-selection marker	forward	miRNA cassette	*GTAGTGAGTCGACCAGTGGATCCTGGAGGCTTGCTGAAGGCTGTATGCTG* ggcctggtgatgatggcgggatcg	65°C
rpslneo counter-selection marker	reverse	miRNA cassette	*ATCTGGGCCATTTGTTCCATGTGAGTGCTAGTAACAGGCCTTGTGTCCTG* tcagaagaactcgtcaagaaggcg	65°C
inserted miRNA oligo	forward	none	ggatcactctcggcatggac	58°C
inserted miRNA oligo	reverse	none	attgccgtcatagcgcgggt	58°C
miRNA-Tom1 (target, **loop,** revTarget)	oligo	miRNA cassette	*GTAGTGAGTCGACCAGTGGATCCTGGAGGCTTGCTGAAGGCTGTATGCTG* TACTGTTCCACGATGGTGTAG<br>**GTTTTGGCCACTGACTGAC**CTACACCAGTGGAACAGTA*CAGGACACAAGGCCTGTTACTAGCACTCACATGG*<br>*AACAAATGGCCCAGAT*	-
miRNA-Tom2 (target, **loop**, revTarget)	oligo	miRNA cassette	*GTAGTGAGTCGACCAGTGGATCCTGGAGGCTTGCTGAAGGCTGTATGCTG* TTGGTGTCCACGTAGTAGTAG<br>**GTTTTGGCCACTGACTGAC**CTACTACTGTGGACACCAA*CAGGACACAAGGCCTGTTACTAGCACTCACATGG*<br>*AACAAATGGCCCAGAT*	-
